# Point-of-Care Ultrasound: A Vital Tool for Anesthesiologists in the Perioperative and Critical Care Settings

**DOI:** 10.7759/cureus.66908

**Published:** 2024-08-14

**Authors:** Ankita Dhir, Dinkar Bhasin, Bhavna Bhasin-Chhabra, Abhilash Koratala

**Affiliations:** 1 Anesthesiology, Max Super Speciality Hospital, Chandigarh, IND; 2 Cardiology, Postgraduate Institute of Medical Education and Research, Chandigarh, IND; 3 Nephrology and Hypertension, Mayo Clinic Arizona, Scottsdale, USA; 4 Nephrology, Medical College of Wisconsin, Milwaukee, USA

**Keywords:** gastric ultrasound, airway ultrasound, perioperative pocus, anaesthesiology, bedside ultrasound, pocus (point of care ultrasound)

## Abstract

Point-of-care ultrasound (POCUS) is an essential skill in various specialties like anesthesiology, critical care, and emergency medicine. Anesthesiologists utilize POCUS for quick diagnosis and procedural guidance in perioperative and critical care settings. Key applications include vascular ultrasound for challenging venous and arterial catheter placements, gastric ultrasound for aspiration risk assessment, airway ultrasound, diaphragm ultrasound, and lung ultrasound for respiratory assessment. Additional utilities of POCUS can include multi-organ POCUS evaluation for undifferentiated shock or cardiac arrest, ultrasound-guided central neuraxial and peripheral nerve blocks, focused cardiac ultrasound, and novel applications such as venous excess ultrasound. This review highlights these POCUS applications in perioperative and intensive care and summarizes the latest evidence of their accuracy and limitations.

## Introduction and background

Point-of-care ultrasound (POCUS) is increasingly being utilized across various acute specialties, including emergency medicine, critical care, and anesthesiology, as a diagnostic and therapeutic tool [1,2]. It includes focused, goal-directed bedside sonographic examinations of various organs, including the lungs, diaphragm, gastric antrum, heart, airway, pelvic organs, and vascular system [3]. Unlike consultative imaging, the physician performing POCUS is responsible for all aspects of image acquisition, interpretation, and formulating the management plan. Incorporating POCUS into routine bedside evaluation has gained substantial recognition, driven by a growing body of evidence demonstrating its superior diagnostic accuracy compared to traditional examination and assessment tools [4]. The perioperative period begins from the patient’s admission, through anesthesia and surgery, to the first 24 hours after the procedure [5]. Anesthesiologists are responsible for patient care and management during this time, making POCUS a vital skill for them. This narrative review summarizes the applications of POCUS in perioperative and intensive care settings, its relevance to anesthesiologists, and the latest evidence related to diagnostic accuracy and validity.

## Review

Vascular ultrasound

Vascular ultrasound is employed for the placement of peripheral and central intravenous catheters and arterial catheters and to identify deep venous thrombosis intraoperatively as well as in critically ill patients.

Peripheral Venous Access and Arterial Line Placement

POCUS proves instrumental in locating deep-seated peripheral veins, which are not readily visible or palpable, including the cephalic, antebrachial, median cubital, and basilic veins [6]. In cases involving patients with difficult intravenous access, ultrasound-guided cannulation demonstrates an impressive success rate, consistently exceeding 90%, compared to the conventional technique, which typically achieves success rates of only 25-30% [7]. This reduces the need for more invasive central venous catheters [8]. These benefits also extend to the pediatric patient population, in whom obtaining peripheral venous access is particularly challenging [9]. Furthermore, POCUS also facilitates the insertion of arterial lines through real-time visualization of landmarks, improving first-pass success rates [10-12]. In a recent randomized controlled trial with 201 participants, arterial line placement was successful on the first attempt in 83.3% of patients in the ultrasound group compared to 55.6% in the digital palpation group (p-value 0.02) [12].

Central Venous Access

**Figure 1:**
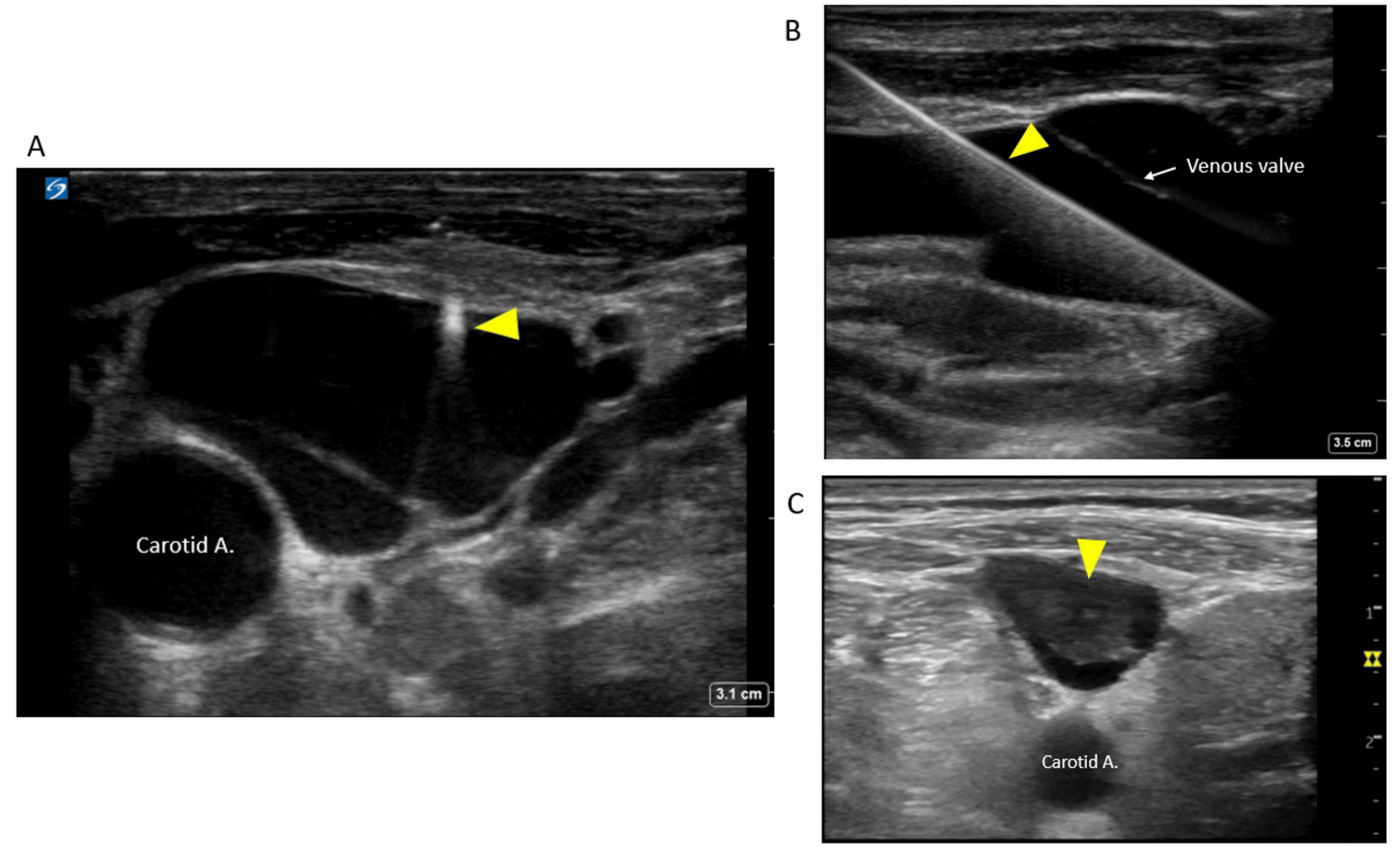
Ultrasound of the internal jugular vein for guiding central venous access placement (A) Needle tip in the vein (arrow). (B) Guidewire in the vein (arrow). (C) Thrombus (arrow) in the internal jugular vein detected prior to catheter placement. Image credit: Abhilash Koratala

The traditional method of obtaining central venous access has been the use of anatomical landmarks to guide needle puncture. However, this method becomes challenging for certain patients with obesity, a history of intravenous drug abuse or chemotherapy, and chronic medical conditions like cancer and peripheral vascular diseases. However, POCUS-guided central venous cannulation is now considered the standard of care [11]. It provides numerous benefits, including preprocedural assessment of vein patency, real-time visualization of the targeted vessel during the procedure, reduced attempts at venipuncture, and the detection of complications such as malpositioned guidewire [13] (Figure 1).

In a systematic review of 35 trials (involving 5,108 participants) comparing ultrasound-guided jugular venous catheter placement to landmark technique, ultrasound guidance increased the success rate from 91.7% to 97.6% (RR: 1.12, 95% CI: 1.08-1.17), enhanced the rate of successful first attempts from 50.1% to 82.2% (RR: 1.57, 95% CI: 1.36-1.82), reduced cannulation time, lowered complication rates from 13.5% to 3.4% (RR: 0.29, 95% CI: 0.17-0.52), and decreased inadvertent arterial punctures from 9.4% to 2.0% (RR: 0.28, 95% CI: 0.18-0.44) [14]. However, a few randomized controlled trials reported that the benefits of ultrasound-guided femoral and subclavian vein (SCV) cannulation did not parallel the improved outcomes seen with internal jugular vein cannulation [15]. Subsequently, a meta-analysis of six randomized controlled trials (953 patients) reevaluated the success and safety of ultrasound-guided SCV catheterization compared to the landmark technique. The real-time ultrasound-guided dynamic approach increased the overall success rate for SCV cannulation, improved the success rate at the first attempt, reduced the total number of attempts, lowered the complication rate, and shortened the time required for a successful procedure [16]. Similar improved outcomes have been shown with the use of ultrasound for femoral vein cannulation as well [11,16].

Deep Vein Thrombosis (DVT)

**Figure 2:**
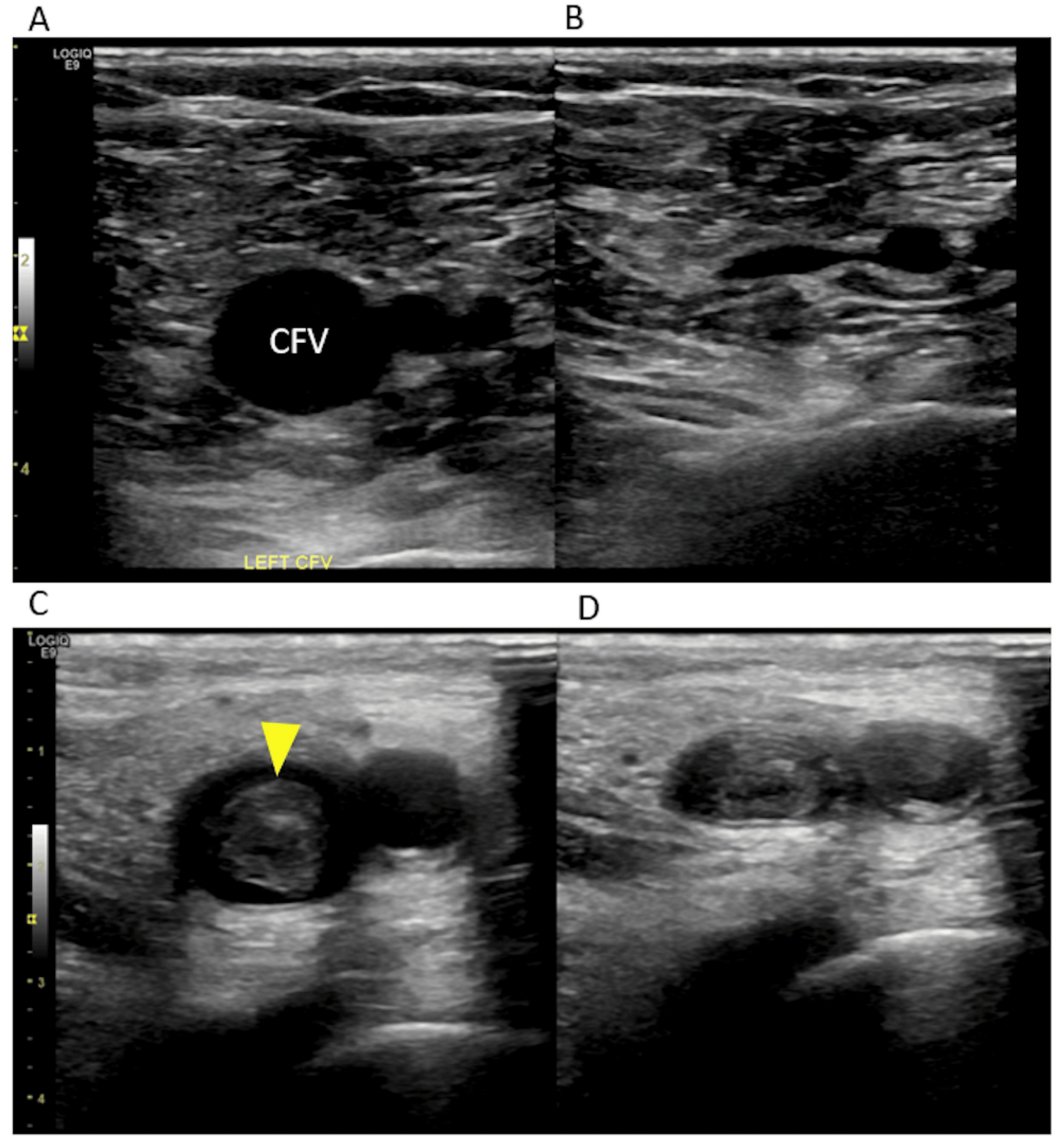
Compression ultrasound for evaluating lower extremity DVT (A) Normal common femoral vein that is fully compressible with transducer pressure (B). (C) Common femoral vein with a hyperechoic thrombus in the lumen (arrow), which is non-compressible (D). DVT: deep vein thrombosis Image credit: Abhilash Koratala

POCUS is a useful tool for the quick bedside diagnosis of lower and upper extremity DVT. This is particularly important in the context of catheter-associated thrombi and suspected cases of acute pulmonary embolism. In a meta-analysis of 43 studies, the sensitivity and specificity to diagnose cases of suspected DVT via proximal compression ultrasound were 90.1% (95% CI: 86.5-92.8) and 98.5% (95% CI: 97.6-99.1), respectively [17]. Figure 2 illustrates a common femoral vein DVT.

Gastric ultrasound

**Figure 3:**
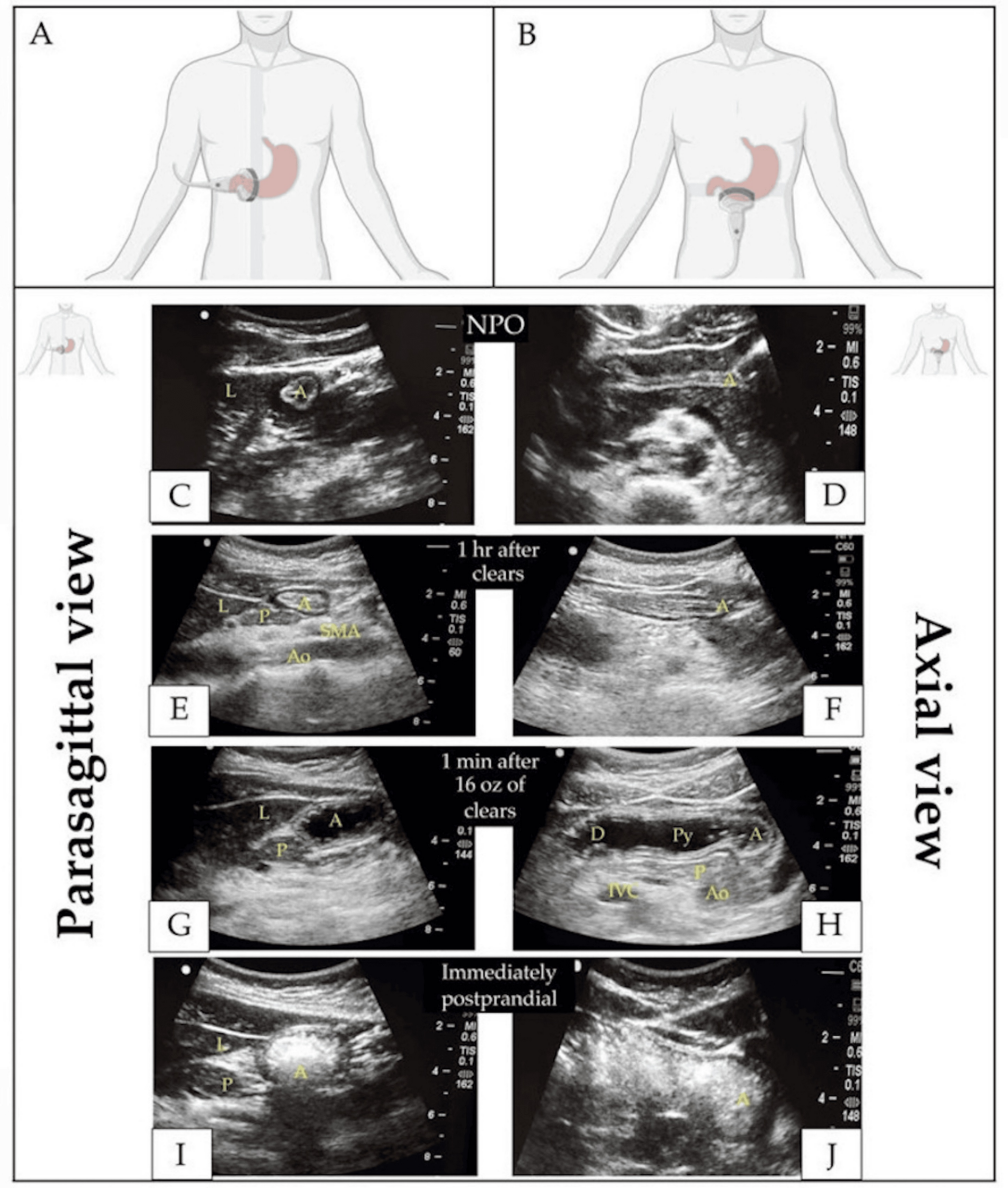
Gastric ultrasound for evaluation of gastric contents (A, B) Ultrasound probe location and orientation for parasagittal and axial views of the gastric antrum. Parasagittal (C, E, G, I) and axial (D, F, H, J) views of the gastric antrum: empty stomach (C, D); one hour after ingestion of clear liquids (E, F); one minute after drinking approximately 500 mL (16 oz) of clear liquids, showing an expanded lumen with hypoechoic contents (G, H); immediately after a meal with a characteristic “ground-glass” appearance (I, J). A: antrum; Ao: aorta; D: duodenum; IVC: inferior vena cava; L: liver; NPO: nil per os; P: pancreas; Py: pylorus; SMA: superior mesenteric artery Created with BioRender.com. Nguyen et al. (2023) [21]; Creative Commons Attribution (CC BY) license

Gastric POCUS facilitates individualized risk assessment of aspiration of gastric contents, which is a major factor contributing to anesthesia-related morbidity and mortality [18,19]. The risk assessment is based on a validated mathematical model established by Perlas et al., which measures the cross-sectional area of the gastric antrum in the right lateral decubitus position [20]. Figure 3 shows the view of the stomach in different states in axial and parasagittal views.

Apart from healthy adults, this model has been tested in various patient populations, including morbidly obese individuals and pregnant patients [22-24]. Gastric POCUS has a sensitivity of 1.0 (95% CI: 0.925-1.0) and a specificity of 0.975 (95% CI: 0-0.072) to detect or rule out a full stomach [25]. POCUS is particularly valuable when the preoperative fasting status is uncertain or in the setting of high aspiration-risk conditions like gastroparesis, diabetic autonomic neuropathy, glucagon-like peptide 1 receptor agonist use, small bowel or gastric outlet obstruction, gastroesophageal reflux disease, increased intraabdominal pressure, and morbid obesity [26,27]. However, gastric ultrasound should only be used as an adjunct rather than a substitute for the standard recommendations for fasting. Further, the ultrasound findings may not be valid in patients with a large hiatus hernia and previous gastric surgery [19].

Gastric ultrasound may also be useful in the intensive care unit to evaluate gastric residual volume following enteral feeding, but it has not been well validated in this setting. While the Perlas mathematical model effectively estimates gastric volume after the intake of clear fluids, ongoing research is focused on developing new models for estimating gastric volume in patients receiving thick enteral feeds [28].

Airway ultrasound 

Identification of the Cricothyroid Membrane (CTM)

**Figure 4:**
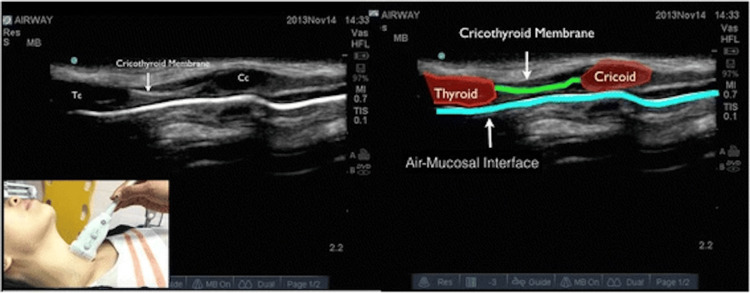
Airway ultrasound for identifying the CTM Cricoid cartilage, thyroid cartilage, and CTM in a longitudinal plane. Cc: cricoid cartilage; CTM: cricothyroid membrane; Tc: thyroid cartilage Osman and Sum (2016) [31]; Creative Commons Attribution (CC BY) license

Surgical cricothyrotomy is a life-saving procedure performed to secure the airway after failed tracheal intubation [29]. Since this procedure is performed infrequently, the conventional method of digital palpation to locate the CTM has more chances of misidentification and failure. POCUS guidance can help improve the accuracy of this procedure [30] (Figure 4).

In a comprehensive meta-analysis conducted by Hung et al., the authors reported a significantly reduced failure rate in CTM identification when employing ultrasound guidance, with a pooled RR of 0.50 (95% CI: 0.33-0.76). Additionally, the study showed a reduced procedural time of 21.8 seconds (95% CI: -1.4 to 45.1), further supporting the efficacy of this technique [32].

Confirmation of Endotracheal Tube (ETT) Placement

**Figure 5:**
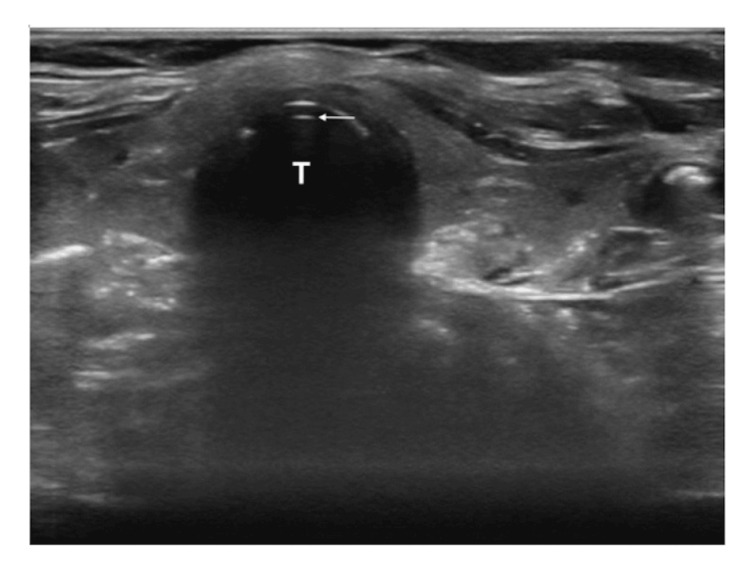
Airway ultrasound showing tracheal intubation The arrow indicates the anterior aspect of the ETT. ETT: endotracheal tube; T: trachea Gottlieb et al. (2024) [35]; Creative Commons Attribution (CC BY) license

**Figure 6:**
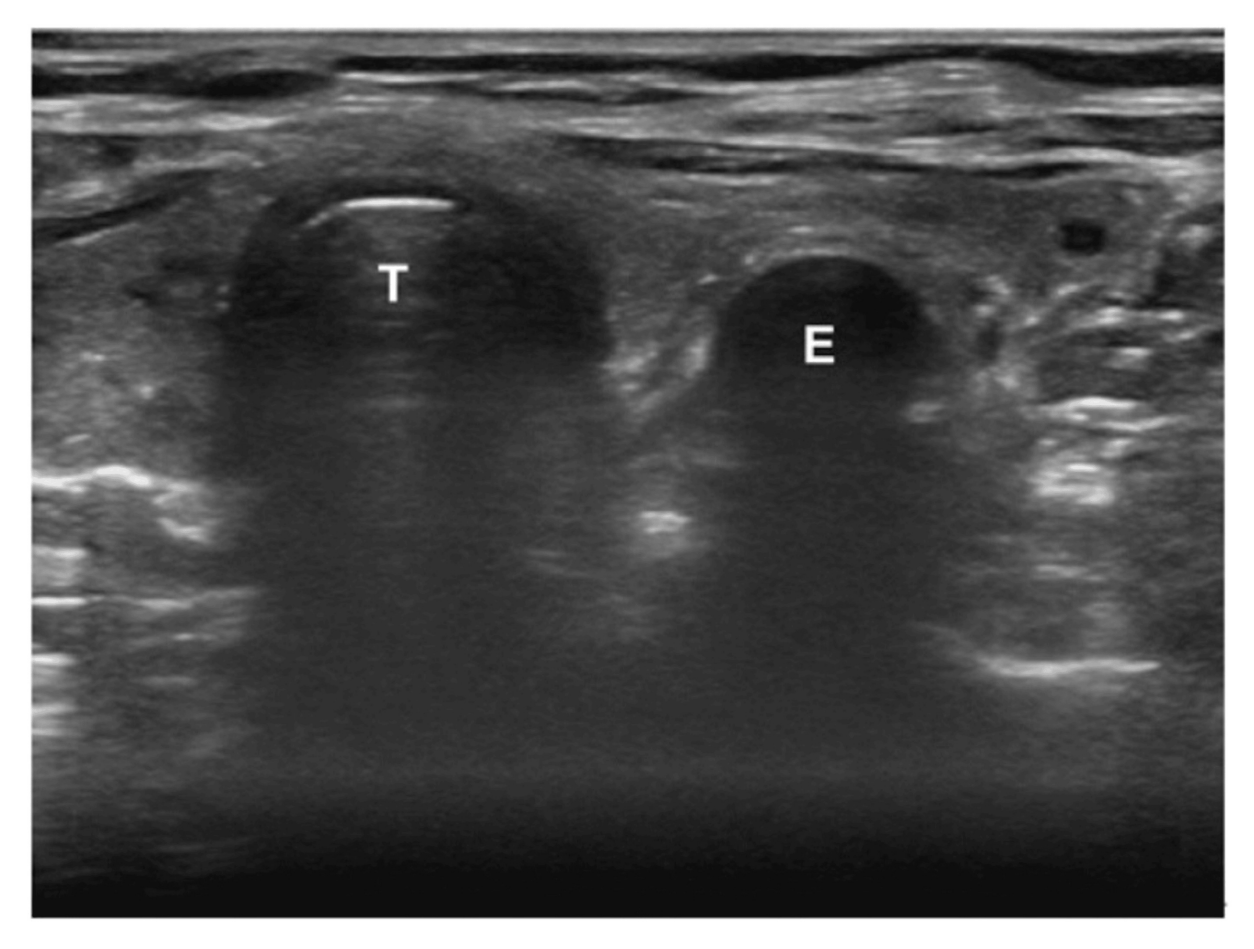
Airway ultrasound showing esophageal intubation In the case of esophageal intubation, a curvilinear structure that mimics the trachea (T) is seen to the right of the trachea. This is the ETT within the esophagus (E) [35]. ETT: endotracheal tube Gottlieb et al. (2024) [35]; Creative Commons Attribution (CC BY) license

Unrecognized esophageal intubation is not uncommon in emergency situations and can lead to serious patient harm [33]. Even when promptly detected, it is associated with a risk of severe hypoxemia, pulmonary aspiration, cardiac arrest, and, in rare instances, gastric or esophageal rupture [34]. Although capnography is considered a reference standard for the verification of ETT, airway POCUS allows easy detection of ETT tube location (Figure 5, Figure 6).

This is particularly useful in certain emergency situations where capnography may become unreliable, like cardiopulmonary arrest, bronchospasm, and pulmonary thromboembolism [36].

Studies have confirmed the high accuracy of tracheal ultrasound in confirming the correct placement of an ETT. In adult patients, it has a sensitivity of 98.7% and a specificity of 97.1%. In pediatric patients, the sensitivity ranges from 92% to 100%, with a consistent specificity of 100% [36,37]. The accuracy remains consistent even in challenging scenarios, such as cardiac arrest [38,39]. In some patients, airway POCUS may be challenging, such as those with a cervical collar, a short neck, or subcutaneous emphysema that extends up to the head and neck [38].

Prediction of a Difficult Airway

Difficult intubation poses a challenge in perioperative management, affecting approximately 4.5-7.5% of cases in the operating room [18]. Predicting difficult intubation becomes particularly complex, and early identification of the risk factors for difficult intubation is crucial to minimizing adverse events.

Traditionally, physical examination of the airway has been the primary method for predicting difficult intubation. This involves evaluations like the modified Mallampati score (with a sensitivity of 53% and a specificity of 80%), the upper lip bite test (with a sensitivity of 67% and a specificity of 92%), and the thyromental distance (with a sensitivity of 37% and a specificity of 89%) [40,41]. Although these methods are valuable, they have their limitations. Airway ultrasound can be used as a complementary tool to clinical assessment in predicting a difficult airway.

Preoperative bedside ultrasound involves measuring distances and ratios between reference points, including the hyomental distance in both neutral position and extension, the skin-to-epiglottis distance (SED), the distance from the skin to the hyoid bone, and the distance from the skin to the vocal cords [31]. A meta-analysis by Benavides-Zora et al. revealed that SED and the hyomental distance measured in extension position exhibited a sensitivity of 75% and 61% and a specificity of 86% and 88%, respectively. Notably, the ratio of the pre-epiglottic distance to the epiglottic distance at the center point of the vocal cords was most accurate in predicting difficult laryngoscopy, with a sensitivity of 82% and a specificity of 83% [42]. Another meta-analysis by Carsetti et al., involving 15 studies, highlighted SED as the most extensively studied parameter for predicting difficult intubation [43]. Hence, adding these tools to routine airway evaluation can significantly improve preoperative assessment.

Other Uses

Airway POCUS finds additional clinical applications, such as aiding in percutaneous tracheostomy by identifying suitable puncture sites and assessing blood vessels [44]. It also assists in determining the appropriate size of a double-lumen tube by measuring the width of the trachea at the sternoclavicular joint [45]. Further, it plays a role in assessing vocal cord function for identifying recurrent laryngeal nerve palsy, as well as detecting tracheal stenosis and tracheal invasion by thyroid cancer [46,47].

Diaphragm ultrasound

The diaphragm is the main inspiratory muscle [48]. Prolonged intubation, the use of muscle relaxants, and sepsis can lead to diaphragmatic muscle weakness in a critically ill patient. Diaphragmatic dysfunction (DD) is defined as a partial (weakness) or complete (paralysis) loss of muscle function, which leads to a reduction in inspiratory capacity and a decrease in respiratory muscle endurance [49]. DD can impact either the hemidiaphragm or both. DD frequently remains undiagnosed in clinical practice due to its nonspecific symptoms, and ultrasound can facilitate an easy bedside assessment of diaphragmatic function [50].

There are two approaches for assessing the diaphragm: the intercostal approach is used to measure parameters like muscle thickness and thickening fraction (TFDi), while the subcostal approach is utilized to gauge diaphragmatic excursion (DE) [51]. Diaphragm muscle weakness is established when DE of less than 10-15 mm during tidal breathing or a maximum TFDi of less than 20% is noted [52]. Diaphragm muscle weakness is a predictor of weaning failure in the ICU and can also be suggestive of increased work of breathing in chronic pulmonary conditions [53]. The diaphragm ultrasound is also useful in cardiac surgery, cervical spine procedures, and after the placement of upper limb blocks (e.g., interscalene blocks) to identify an iatrogenic phrenic nerve injury [51].

In a meta-analysis conducted by Parada et al., including 19 studies (1,204 participants), the sensitivity of diaphragm ultrasound for evaluating diaphragm excursion was 0.80 (95% CI: 0.77-0.83), while the specificity was 0.80 (95% CI: 0.75-0.84). Regarding the assessment of the diaphragm thickening fraction, the sensitivity was 0.85 (95% CI: 0.82-0.87), while the specificity was 0.75 (95% CI: 0.69-0.80) [54]. Although diaphragm ultrasound is reliable, there remain issues related to standardization because of the significant variability in image acquisition and methodology [55].

Lung ultrasound (LUS)

LUS is a valuable bedside diagnostic tool for assessing various respiratory pathologies [56,57]. Although LUS cannot directly image lung tissue as the air in the lungs scatters the ultrasound beam, the interpretation of artifacts and specific patterns can aid in the diagnosis [58]. It exhibits better diagnostic accuracy than auscultation and chest radiography in detecting pleural effusion, pneumothorax, acute respiratory distress syndrome (ARDS), and cardiogenic pulmonary edema [59]. According to a meta-analysis of nine studies, for detecting cardiogenic pulmonary edema, the sensitivity of LUS was 0.92 (95% CI: 0.84-0.97), and the specificity was 0.87 (95% CI: 0. 82-0.91) [60]. Another meta-analysis conducted by Ding et al. showed that LUS had more sensitivity (0.88) compared to chest X-rays (0.52) with the same specificity for the diagnosis of pneumothorax [61].

Normal LUS shows a shimmering (sliding) hyperechoic pleural line followed by horizontal reverberation artifacts, parallel to the pleural line, known as the A-lines [62,63]. Absent pleural sliding should raise suspicion for pneumothorax since the air in between the pleural layers abolishes sliding. However, visualizing the junction where the pleura transitions from normal sliding to absent sliding, known as the “lung point,” is more specific [62]. Effusions are identified as anechoic collections between the parietal and visceral pleura, first appearing in the dependent zone and then in other regions when extensive [64].

**Figure 7:**
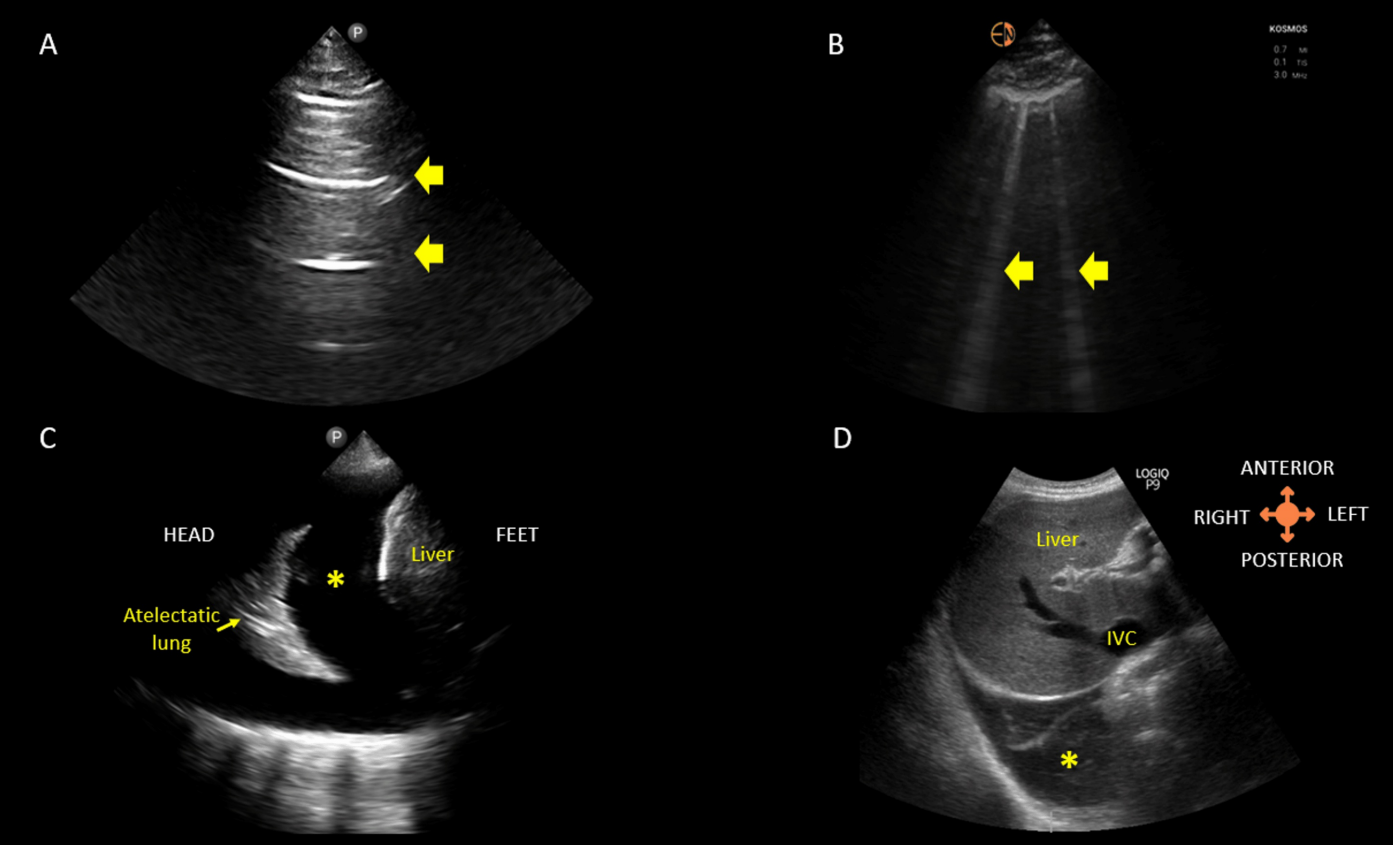
LUS (A) Normal lung showing horizontal artifacts, i.e., A-lines (arrows). (B) Vertical artifacts (arrows) known as B-lines indicate interlobular septal thickening, typically seen in congestion. (C) Pleural effusion (asterisk) as seen on a lateral scan. (D) Right pleural effusion (asterisk) as seen from the subxiphoid scanning window. IVC: inferior vena cava; LUS: lung ultrasound Turk et al. (2023) [66]; Creative Commons Attribution (CC BY) license

The presence of vertical hyperechoic artifacts, known as B-lines, that move with pleural sliding is the hallmark of interstitial syndrome. The interstitial syndrome includes any pathological condition leading to increased density in the interstitial space between alveoli. This includes pulmonary edema, pneumonia, ARDS, COVID-19, or pulmonary fibrosis [65]. In consolidation, the lung tissue appears like the liver (also called hepatization), often surrounded by some pleural effusion. Atelectasis appears similar to consolidation, and clinical context helps in differentiating these two conditions [64]. Some signs, such as mobile air in the airways (dynamic air bronchograms), favor consolidation over atelectasis. Some common ultrasonographic signs seen in lung pathologies are shown in Figure 7.

POCUS for the evaluation of patients with undifferentiated shock and cardiac arrest

Perioperative cardiac arrest is a rare but potentially catastrophic event. According to the UK-wide prospective 7th National Audit Project (NAP7), the incidence of perioperative cardiac arrest is approximately three in 10,000, mostly occurring during non-elective, complex surgeries, while the incidence of potentially serious complications is one in 18 (6%) cases [67]. POCUS in undifferentiated shock or cardiac arrest can identify treatable causes (such as hypovolemia, hypoxia, cardiac tamponade, pneumothorax, and pulmonary embolism), assess the quality of chest compressions during CPR, and differentiate true pulseless electrical activity (PEA) from pseudo-PEA [68]. It can also provide prognostic information regarding the possibility of a return to spontaneous circulation and survival [69]. Established protocols like RUSH, POCUS-CA, SHoc-ED, and FATE offer algorithmic frameworks for sonographic assessment [68,70-72]. Yoshida et al. conducted a meta-analysis of 12 studies with 1132 patients and concluded that the sensitivity and specificity of POCUS in determining the type of shock were 0.82 and 0.98 for obstructive shock, 0.78 and 0.96 for cardiogenic shock, 0.90 and 0.92 for hypovolemic shock, and 0.79 and 0.96 for distributive shock, respectively [73]. The current cardiopulmonary resuscitation guidelines recommend that POCUS be considered an extra diagnostic tool in cases where experienced personnel can perform it without disrupting CPR, particularly when there is a clinical suspicion of a specific reversible cause [74].

POCUS for regional anesthesia

Neuraxial Ultrasound 

Central neuraxial blocks (CNBs), such as spinal, epidural, and combined spinal epidural blocks, depend on surface anatomical landmarks, tactile perception, and optimal patient positioning for procedural success. Hence, these blocks can be challenging for certain patient subsets, such as obese patients, the elderly, pregnant patients, and those with spinal deformities like scoliosis [75,76].

**Figure 8:**
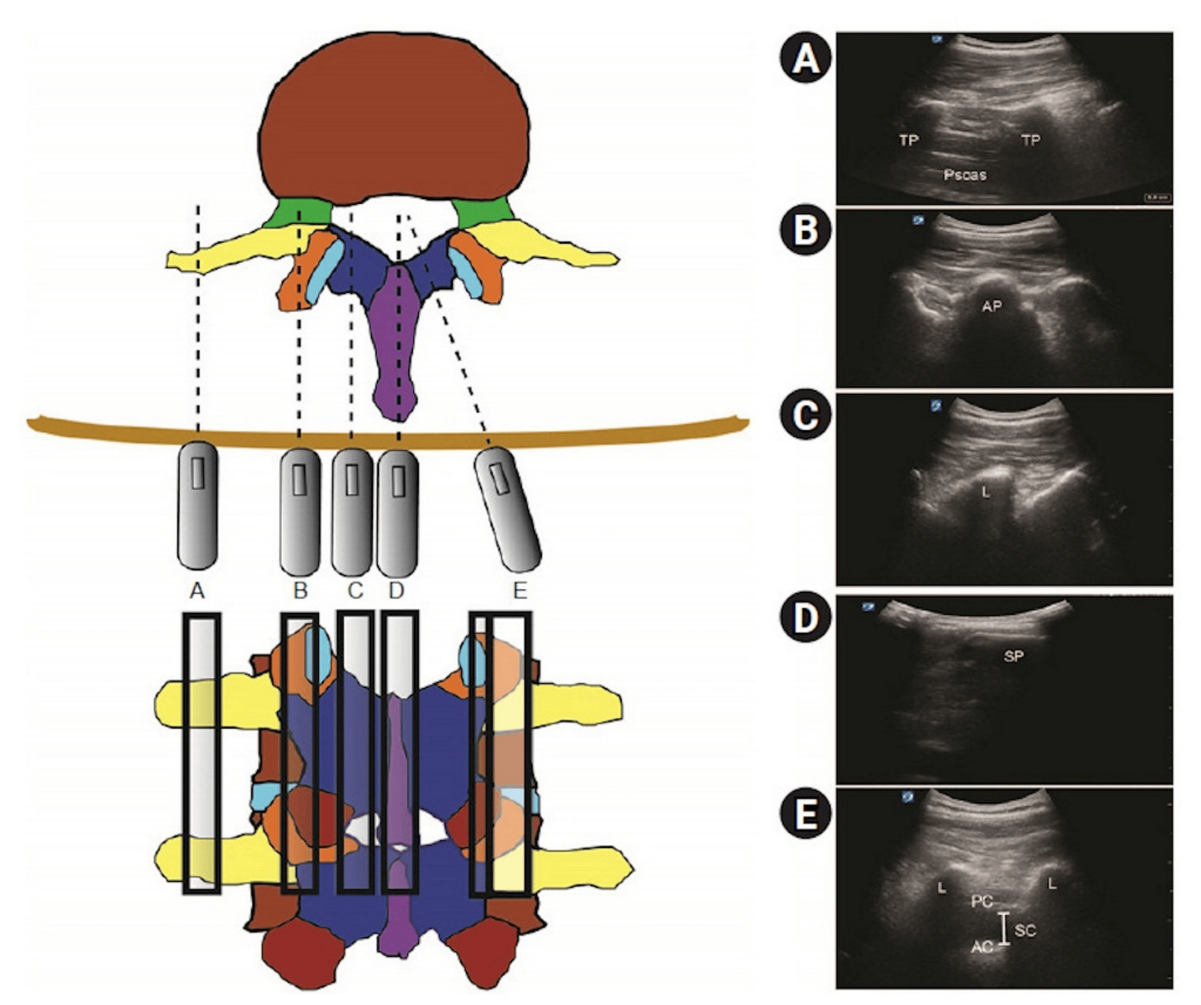
Sagittal views of the lumbar spine (A) Sagittal transverse process view. (B) Sagittal articular process view. (C) Sagittal lamina view. (D) Sagittal spinous process view. (E) Parasagittal oblique view. AC: anterior complex; AP: articular process; L: lamina; PC: posterior complex; SC: spinal canal (intrathecal space); SP: spinous process; TP: transverse process Yoo et al. (2020) [79]; Creative Commons Attribution (CC BY) license

**Figure 9:**
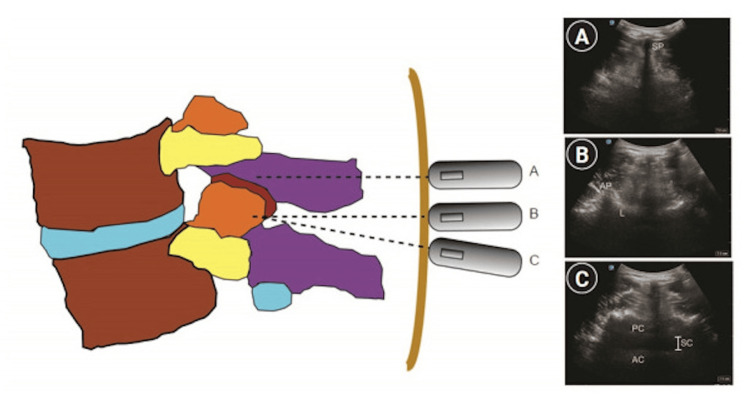
Transverse views of the lumbar spine (A) Transverse spinous process view. (B) Transverse interspinous process view. (C) Tilted transverse interspinous process view. AC: anterior complex; AP: articular process; L: lamina; PC: posterior complex; SC: spinal canal (intrathecal space); SP: spinous process Yoo et al. (2020) [79]; Creative Commons Attribution (CC BY) license

Ultrasound has become a valuable tool for performing safe CNBs by providing real-time images of the spinal anatomy to guide the procedure. It is useful in pre-procedural scanning, aiding in midline localization, identification of intervertebral spaces, depth measurement, and anticipation of potential challenges [77-79]. It can also guide real-time needle placement, improve success rates, and enhance patient comfort [80]. However, image acquisition skills for neuraxial ultrasound have a steep learning curve and may be especially challenging in elderly patients with calcifications and obese patients [79]. Figure 8 and Figure 9 show the transverse and sagittal views of the spine required for conducting neuraxial ultrasound.

Ultrasound-Guided Regional Nerve Blocks 

Ultrasound guidance for performing regional nerve blocks is now the standard of care. It facilitates real-time visualization of neural structures, monitoring needle placement, and assessing the spread of the local anesthetic agent. Compared to the traditional landmark method, ultrasound-guided blocks have higher success rates, decreased procedure times, a lesser anesthetic agent dose requirement, and a lower incidence of inadvertent vascular punctures and complications like pneumothorax [81-85].

In a comprehensive meta-analysis of 23 trials with more than 2,000 peripheral nerve blocks, the utilization of ultrasound guidance, either in isolation or in addition to nerve stimulation, showed a significant decrease in the incidence of vascular puncture, decreased procedural pain, and a lower requirement for additional analgesia or anesthesia [86]. However, there was no reduction in the occurrence of postoperative neurological complications. A recent consensus statement strongly advocates the utilization of ultrasound for regional anesthesia, supported by a high level of certainty in the available evidence [87].

Focused cardiac ultrasound (FoCUS) and hemodynamic assessment

FoCUS is a valuable tool in evaluating hemodynamically unstable patients in the perioperative period and intensive care [88,89]. Unlike formal transthoracic echocardiography (TTE), FoCUS aims at a point-of-care, limited cardiac evaluation that quickly recognizes specific ultrasound signs, which can help in narrowing down the differential diagnosis. The competence achieved in FoCUS can either be basic critical care echocardiography (CCE) or advanced CCE, depending on the training received [90].

**Figure 10:**
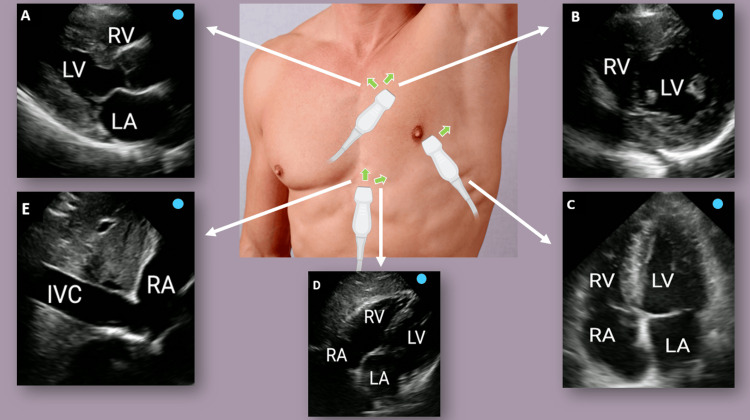
Basic echocardiographic views (A) Parasternal long axis. (B) Parasternal short axis. (C) Apical four-chamber. (D) Subxiphoid; (E) IVC. The green arrows indicate the direction of the transducer orientation marker. IVC: inferior vena cava; LA: left atrium; LV: left ventricle; RA: right atrium; RV: right ventricle Argaiz et al. (2021) [91]; reproduced with permission from Wolters Kluwer Health

Basic CCE utilizes standard two-dimensional TTE and generally excludes the use of spectral Doppler applications. Commonly used transthoracic views for this purpose are shown in Figure 10.

**Figure 11:**
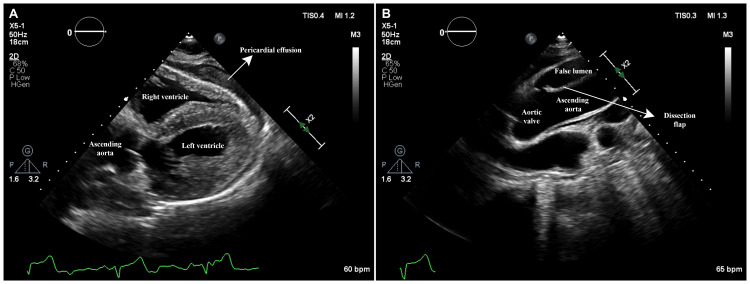
Ascending aortic dissection with cardiac tamponade Transthoracic echocardiogram in a patient with ascending aortic dissection and cardiac tamponade: (A) Subcostal view showing a large pericardial effusion causing RV collapse. (B) A high-right parasternal view demonstrating the intimal dissection flap in the ascending aorta. RV: right ventricular Image credit: Dinkar Bhasin

It involves assessment of overall left ventricular (LV) function and size, appreciation of regional wall motion abnormalities, identification of pericardial effusion, evaluation of right ventricular (RV) function and size, and detection of significant valvular lesions based on color Doppler. Basic CCE is aimed at rapidly categorizing shock states and identifying life-threatening causes such as hypovolemic shock, cor pulmonale, possible aortic dissection, and cardiac tamponade [90,92] (Figure 11).

**Figure 12:**
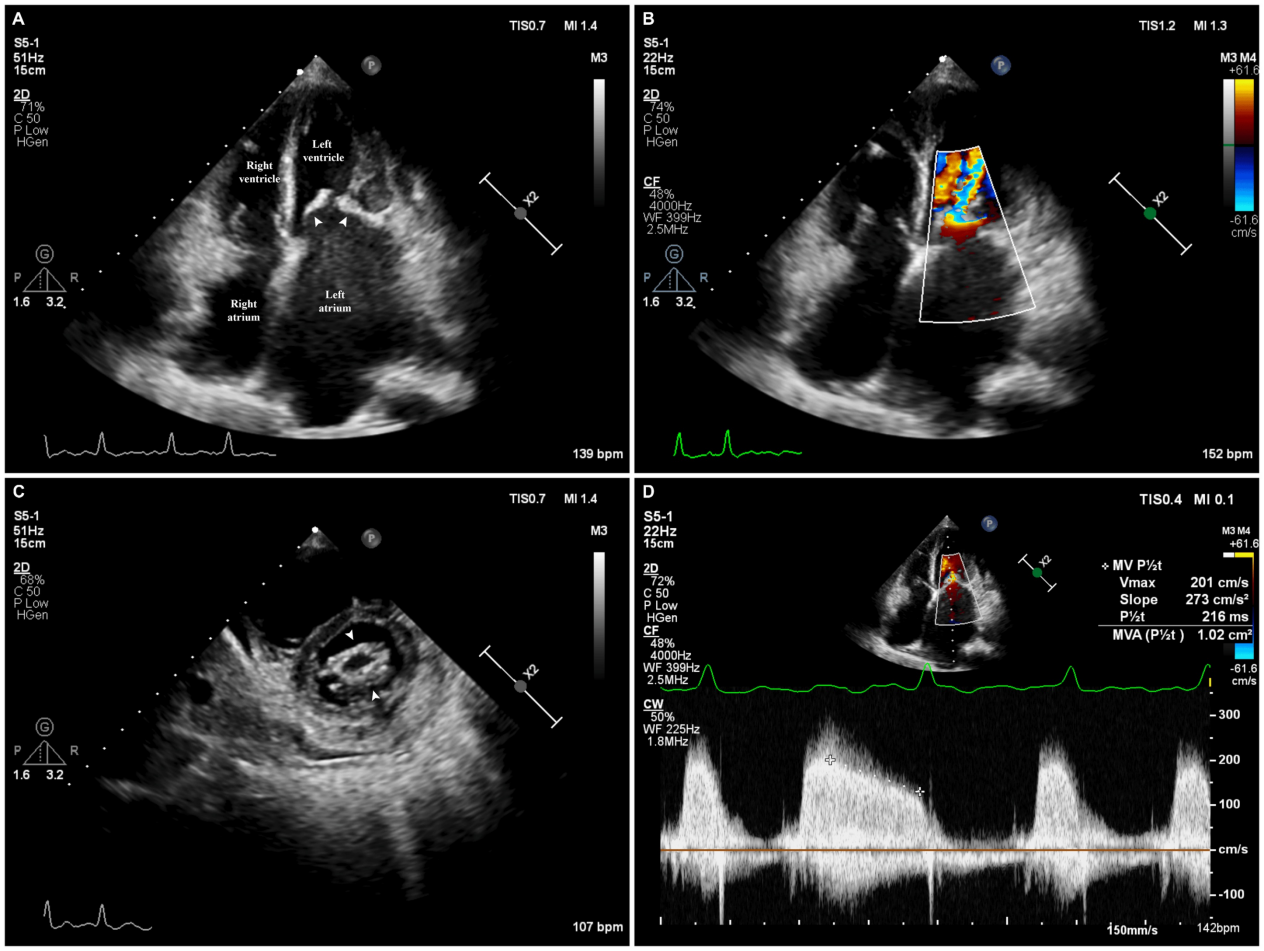
Severe mitral stenosis in a patient with shock (A) Apical four-chamber view demonstrates thickened mitral valve leaflets and a dilated left atrium (arrowheads). (B) Color Doppler imaging across the mitral valve shows turbulent flow across the mitral valve in diastole. (C) Parasternal short-axis view demonstrating thickened mitral valve leaflets (arrowheads) with commissural fusion giving a fish-mouth appearance. Mild pericardial effusion can also be appreciated. (D) Continuous-wave Doppler across the mitral valve demonstrates an elevated gradient. The valve area, as measured by pressure half-time, is 1 cm^2^. Image credit: Dinkar Bhasin

Advanced CCE provides a more comprehensive and quantitative assessment of cardiac function, including parameters like cardiac output, valvular pathology, diastolic dysfunction, and pulmonary hypertension, for optimizing hemodynamics at the bedside (Figure 12).

In a meta-analysis of nine studies comparing the accuracy of clinical assessment with FOCUS for diagnosing various conditions, FoCUS-based examination was more sensitive (84% vs. 43%) and specific (89% vs. 81%) compared to clinical assessment for identifying LV dysfunction (LV ejection fraction <50%) [91]. Furthermore, FOCUS-based examination had a higher sensitivity (71% vs. 46%) for diagnosing aortic or mitral valve disease (of at least moderate severity) compared to clinical examination, with both having a similar specificity of 94% [93]. While not intended to replace existing diagnostic methods, FOCUS complements traditional tools and exams that may miss important cardiac diagnoses [94]. In patients with difficult transthoracic windows and during cardiopulmonary resuscitation, critical care transesophageal echocardiography (TEE) is emerging as a valuable bedside tool [95].

Some practical tips for performing FoCUS can be useful. For critically ill patients, particularly those on mechanical ventilation, obtaining good transthoracic views can be challenging. The subcostal window provides good alternative views for assessing ventricular function and valvular lesions. The plane of the subcostal four-chamber view is similar to the apical four-chamber view and gives an idea of the global LV function. Rotating the echo probe counter-clockwise from the subcostal four-chamber view yields short-axis views similar to the parasternal short-axis view and can help identify regional wall motion abnormalities. The subcostal four-chamber view is also good for identifying pericardial effusions and the RV collapse in cardiac tamponade. While TTE can facilitate early diagnosis of ascending aortic dissection by demonstrating a dissection flap, the diagnostic yield is poor, and aortic dissection should not be ruled out based on a negative TTE alone. The yield of TTE can be improved by employing high parasternal views, such as the right parasternal views, as illustrated in Figure 6. However, when clinical suspicion is high, TEE, or computed tomography, should be considered.

**Figure 13:**
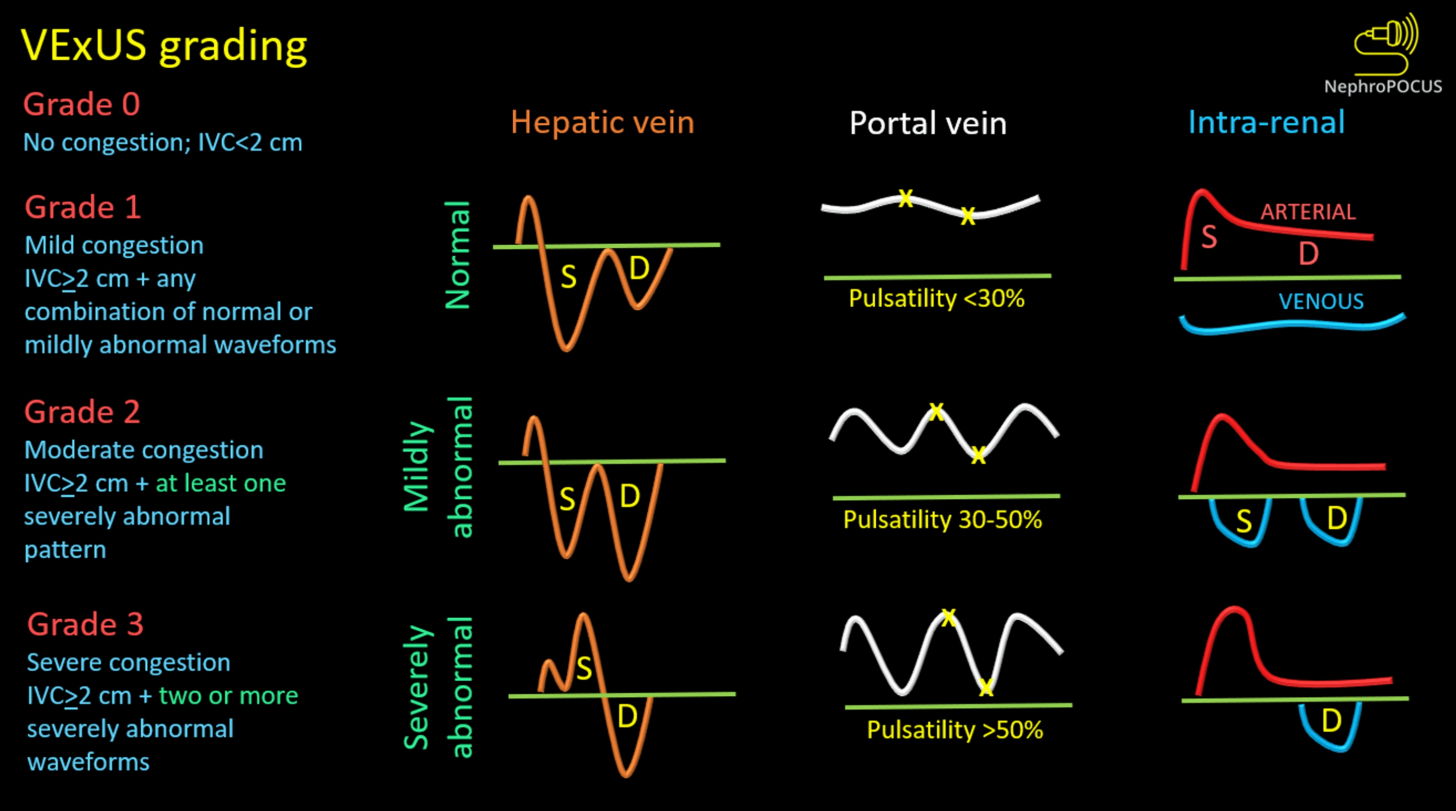
VExUS grading When the diameter of the IVC is >2 cm, three grades of congestion are defined based on the severity of abnormalities on the hepatic, portal, and renal parenchymal venous Doppler. Hepatic vein Doppler is considered mildly abnormal when the systolic (S) wave is smaller than the diastolic (D) wave but still below the baseline; it is considered severely abnormal when the S-wave is reversed. Portal vein Doppler is considered mildly abnormal when the pulsatility is 30-50%, and severely abnormal when it is ≥50%. Asterisks represent points of pulsatility measurement. Renal parenchymal vein Doppler is mildly abnormal when it is pulsatile with distinct S and D components and severely abnormal when it is monophasic with a D-only pattern. IVC: inferior vena cava; VExUS: venous excess ultrasound Adapted from NephroPOCUS.com with permission

In recent times, there has been a growing awareness of the harmful effects of fluid overload, particularly in critical illness scenarios where the empirical use of intravenous fluids is prevalent [96]. A systematic review including 19,902 patients admitted to the intensive care unit revealed that non-survivors had a cumulative fluid balance 4.4 L higher than survivors after one week of ICU stay. Additionally, adopting a restrictive fluid management approach was linked to lower mortality rates compared to the outcomes associated with liberal fluid administration [97]. A novel sonographic assessment known as venous excess ultrasound (VExUS) is gaining prominence as a method to grade systemic venous congestion and monitor response to decongestive therapy [98-100]. The VExUS protocol combines the use of inferior vena cava (IVC) ultrasound with pulsed-wave Doppler assessment of the hepatic, portal, and intrarenal veins to generate a numerical score, as illustrated in Figure 13.

These IVC measurements and Doppler scans help assess the degree of venous congestion, categorizing it as mild, moderate, severe, or none. VExUS can be a valuable adjunct to bedside hemodynamic assessment as a noninvasive, individualized method to optimize fluid management in surgical and critically ill patients. In a group of post-cardiac surgery patients, the identification of flow abnormalities in two or more veins (among hepatic, portal, and kidney parenchymal veins) along with a full or dilated IVC (≥2 cm) has demonstrated the ability to predict the risk of acute kidney injury with greater accuracy (HR: 3.69; 95% CI: 1.65-8.24; p = 0.001) compared to relying solely on isolated central venous pressure measurements [101]. Clinical trials studying the utility of VExUS in various critically ill subgroups are currently underway [102].

## Conclusions

POCUS is an essential skill for anesthesiologists in perioperative and critical care settings. It not only complements the traditional physical examination for accurate assessment of the patient but also enhances the procedural success rates of various invasive procedures where ultrasound guidance can be employed. In the preoperative period, airway ultrasound can be used to anticipate challenging airways, and gastric ultrasound can be utilized for aspiration risk assessment. During the intraoperative period, ultrasound can be utilized to perform procedures like peripheral and central vascular access, arterial line placement, and central neuraxial and peripheral nerve blocks. Multi-organ POCUS can provide useful clues in cases of perioperative emergencies or peri-arrest states. In the postoperative period or in intensive care, VExUS, FoCUS, and LUS may be helpful in the diagnosis of any pulmonary or cardiac complications and the assessment of fluid status. The latest evidence supports that POCUS leads to better quality of care and patient outcomes. As evidence of its benefits continues to grow, the use of POCUS in patient management is set to increase, making it essential to include POCUS in the training programs and curricula for anesthesiologists.
